# Tailoring Mobile Data Collection for Intervention Research in a Challenging Context: Development and Implementation in the Malakit Study

**DOI:** 10.2196/29856

**Published:** 2022-06-16

**Authors:** Yann Lambert, Muriel Galindo, Martha Suárez-Mutis, Louise Mutricy, Alice Sanna, Laure Garancher, Hedley Cairo, Helene Hiwat, Jane Bordalo Miller, José Hermenegildo Gomes, Paola Marchesini, Antoine Adenis, Mathieu Nacher, Stephen Vreden, Maylis Douine

**Affiliations:** 1 Centre d’Investigation Clinique Antilles-Guyane Institut national de la santé et de la recherche médicale (Inserm 1424) Centre Hospitalier de Cayenne Andrée Rosemon Cayenne French Guiana; 2 Parasitic Disease Laboratory Institute Oswaldo Cruz Foundation Oswaldo Cruz Rio de Janeiro Brazil; 3 The Ink Link Paris France; 4 Malaria Program Ministry of Health of Suriname Paramaribo Suriname; 5 Desenvolvimento, Prevenção, Acompanhamento e Cooperação de Fronteiras Oiapoque Brazil; 6 Malaria Technical Group Vector Transmissible and Zoonotic Diseases Coordination Ministry of Health Brasilia Brazil; 7 Foundation for Scientific Research Suriname Paramaribo Suriname

**Keywords:** malaria, Guiana Shield, information system, mobile data collection, Open Data Kit, ODK

## Abstract

**Background:**

An interventional study named Malakit was implemented between April 2018 and March 2020 to address malaria in gold mining areas in French Guiana, in collaboration with Suriname and Brazil. This innovative intervention relied on the distribution of kits for self-diagnosis and self-treatment to gold miners after training by health mediators, referred to in the project as facilitators.

**Objective:**

This paper aims to describe the process by which the information system was designed, developed, and implemented to achieve the monitoring and evaluation of the Malakit intervention.

**Methods:**

The intervention was implemented in challenging conditions at five cross-border distribution sites, which imposed strong logistical constraints for the design of the information system: isolation in the Amazon rainforest, tropical climate, and lack of reliable electricity supply and internet connection. Additional constraints originated from the interaction of the multicultural players involved in the study. The Malakit information system was developed as a patchwork of existing open-source software, commercial services, and tools developed in-house. Facilitators collected data from participants using Android tablets with ODK (Open Data Kit) Collect. A custom R package and a dashboard web app were developed to retrieve, decrypt, aggregate, monitor, and clean data according to feedback from facilitators and supervision visits on the field.

**Results:**

Between April 2018 and March 2020, nine facilitators generated a total of 4863 form records, corresponding to an average of 202 records per month. Facilitators’ feedback was essential for adapting and improving mobile data collection and monitoring. Few technical issues were reported. The median duration of data capture was 5 (IQR 3-7) minutes, suggesting that electronic data capture was not taking more time from participants, and it decreased over the course of the study as facilitators become more experienced. The quality of data collected by facilitators was satisfactory, with only 3.03% (147/4849) of form records requiring correction.

**Conclusions:**

The development of the information system for the Malakit project was a source of innovation that mirrored the inventiveness of the intervention itself. Our experience confirms that even in a challenging environment, it is possible to produce good-quality data and evaluate a complex health intervention by carefully adapting tools to field constraints and health mediators’ experience.

**Trial Registration:**

ClinicalTrials.gov NCT03695770; https://clinicaltrials.gov/ct2/show/NCT03695770

## Introduction

*Plasmodium falciparum* and *Plasmodium vivax* malaria remain endemic in the Region of the Guiana Shield [1]. In French Guiana, the main reservoir of the parasites is the population of 10,000 to 15,000 gold miners working clandestinely in the Amazon forest [2]. Mostly originating from Brazil, they have virtually no access to health care while working on the mining sites [3]. In case of malaria-like symptoms, they frequently use under-the-counter antimalarials, without diagnosis and with poor adherence. This behavior, in combination with high mobility within the forest and across borders, may jeopardize the efforts to control malaria in the region of the Guiana Shield, paving the way for the emergence of antimalarial-resistant *Plasmodium* parasites [4,5]. To address this concern, French Guiana, Brazil, and Suriname jointly implemented an interventional study called Malakit from April 2018 to March 2020. This innovative collaborative project investigated the feasibility and effectiveness of distributing kits for self-diagnosis and self-treatment to gold miners after being trained on how to use them by health mediators [6,7].

Evaluating the effectiveness and transferability of such a novel strategy is always crucial, but the complex environment in which Malakit was implemented made this even more challenging [8]. The evaluation of the Malakit strategy was based on (1) the comparison of before-and-after cross-sectional estimates of appropriate behavior of gold miners with regard to malaria care and (2) the longitudinal monitoring of participants’ adherence to correct kit use and safety. This pragmatic study design was chosen to ensure feasibility in this particular context and because the limited level of evidence produced needed support from data of the best possible quality. An information system was, thus, developed to address strong field constraints, while meeting ethical and regulatory standards, with a mobile health (mHealth) approach to data capture, management, and monitoring.

In this paper, we describe the process by which the Malakit information system was designed, developed, and implemented to achieve the monitoring and evaluation of the Malakit intervention. Through quantitative and qualitative feedback, we present our experience with mobile data collection (MDC) tools and how they may be used successfully by health mediators, hereafter referred to as facilitators.

## Methods

### Context and Needs of the Malakit Study

#### Field Constraints

French military forces are fighting illegal gold mining to limit its expansion in French Guiana. Clandestine gold miners enter the French territory through crossing points at the borders with Brazil and Suriname. These “resting sites” are strategically located in Brazil and Suriname, outside the borders of French Guiana and beyond the reach of the French military forces. They provide logistical and economical support to the gold mining system [5].

**Figure 1:**
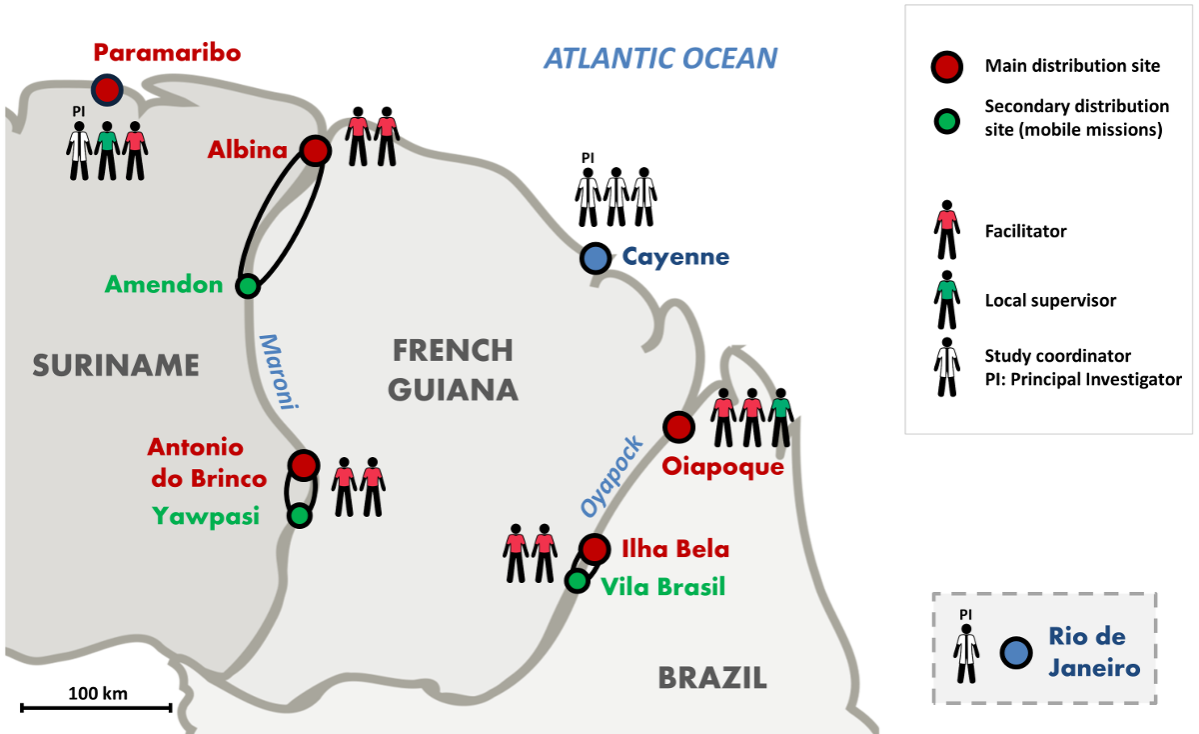
Distribution sites and staff of the Malakit study, Guiana Shield, April 2018-March 2020.

The Malakit intervention relied on facilitators who were responsible for the distribution of kits and the training of participants. These facilitators were chosen for their knowledge of the gold miners’ community and their ability to communicate with them. The hiring, training, supervision, and different missions of Malakit facilitators are detailed in previous articles [7,9]. Kits were distributed at four fixed resting sites along the borders of French Guiana, with the continuous presence of two facilitators. One additional distribution site was set up in Anamoestraat, an area well frequented by Brazilian gold miners in Paramaribo, the capital city of Suriname. Furthermore, occasional mobile missions were organized at three secondary distribution sites selected for their strategic importance for the gold miners’ movements to and from mining areas in French Guiana [6,7]. In Brazil and Suriname, facilitators received administrative and logistical support from one local supervisor who was in contact with the study coordination team in Cayenne. Figure 1 shows the location of the distribution sites in Suriname and Brazil, and the staff involved in the Malakit study. As described in Table 1, the geographical and logistical context varied significantly among distribution sites and impacted the work of facilitators. The Malakit information system needed to take these local constraints into account.

**Table 1:** Geographical and logistical context of distribution sites in the Malakit study, Guiana Shield, April 2018-March 2020.

Location and distribution site			Distribution mode		Environment		Access		Internet bandwidth		Electrical supply		Number of facilitators^a^
**Maroni River, Surinamese border**													
	Albina	Fixed		Urban		Road, boat		Good		Good		2	
	Amendon	Mobile mission		Forest, isolated		Boat only		None		None		1 (Albina)	
	Antonio do Brinco	Fixed		Urban, isolated		Plane, boat		Poor		Evening only		2	
	Yawpasi	Mobile mission		Forest, isolated		Plane, boat		None		None		1 (Antonio do Brinco)	
**Suriname, capital city**													
	Paramaribo	Fixed		Urban		Road, plane		Excellent		Excellent		1	
**Oyapock River, Brazilian border**													
	Oiapoque	Fixed		Urban		Road, boat, plane		Good		Good, occasional power cuts		2	
	Ilha Bela	Fixed		Forest, very isolated		Boat only		None		Evening only		2	
	Vila Brasil	Mobile mission		Urban, very isolated		Boat only		Poor		Evening only		2 (Ilha Bela)	

^a^The origin of the facilitators involved in mobile missions at secondary distribution sites is shown in parentheses.

#### Prototyping of the Information System

The gold mining community actively contributed to the development of Malakit. A participatory approach helped to prototype and validate the contents of the intervention (ie, kit design, training steps, and training materials, such as illustrations and videos) during multiple field missions at the resting sites in 2017 and early 2018 with groups of gold miners and key actors of the community [7,10].

**Figure 2:**
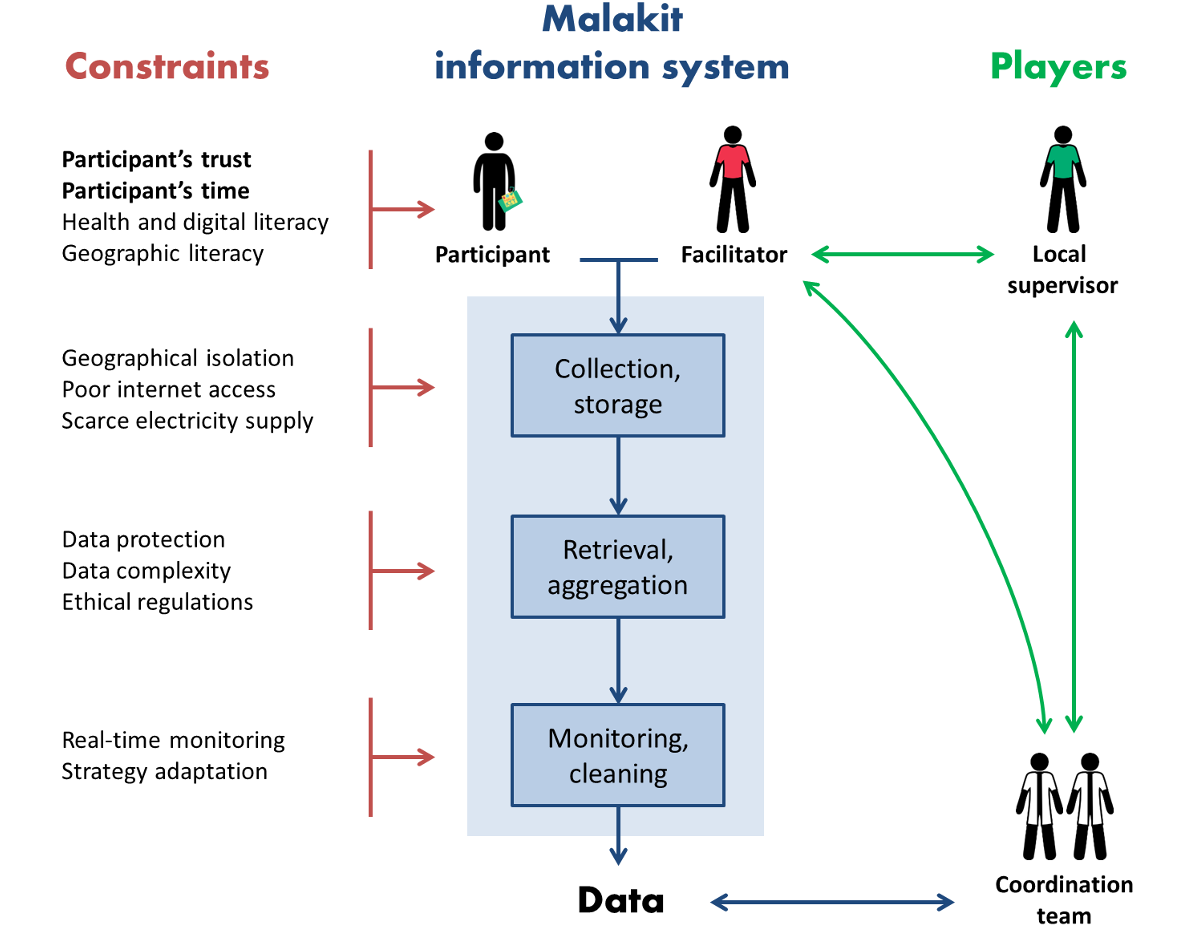
Constraints influencing the design of the Malakit information system (Malakit study, Guiana Shield, April 2018-March 2020).

The information system involved participants of the study, nine facilitators, two local supervisors, and the study coordination team. The principal investigators of the study were located in Brazil, Suriname, and French Guiana. The information system development and maintenance relied on one member of the coordination team in Cayenne, in close relationship to the study sponsor. The interaction of these different players in the Malakit project was another source of complexity added to the heterogeneity of the settings of distribution sites, as illustrated by the variety of languages used for communication (ie, Portuguese, English, French, and Dutch). Facilitators showed varying levels of health, digital, and geographic literacy, and little to no experience in participant enrollment and data collection [7]. To ensure the quality of the data and allow for real-time monitoring, the design of the Malakit information system had to adapt to these multiple constraints. Figure 2 shows the influence of these constraints on each component of the information system and on its users.

Participants’ trust and available time were regarded as the most critical constraints to take into account. Gold miners going to French Guiana wait for any opportunity to cross the border and have little time to spend at resting sites. In addition, because of their clandestine status, gold miners may be wary of any action promoted by the French authorities, and data collection for research may be confused with police intelligence. It was, therefore, very important throughout the study to be transparent about the use of the data. The hiring of facilitators from the gold mining community as an interface between the coordination team and the participants helped establish a climate of trust. Any communication about the study clearly stated its objectives and the purpose of data collection. Several components of the information system were designed to preserve this trust: participants’ anonymization, questionnaire design (eg, no questions regarding future destinations and freedom to not answer), and data encryption. Before the start of the study, a first version of the information system was presented to the scientific committee of the Malakit project, which included one facilitator with several years of experience as a community health worker in the Malaria Service Deliverer network in Suriname. She helped rephrase questions, helped identify questions that could appear or would likely appear sensitive to the participants, and insisted on keeping the questionnaires as short as possible to avoid wasting time.

#### Paperless Data Collection and Digital Device Selection

The choice of electronic data capture (EDC) over paper was obvious. The simultaneous collection of longitudinal data from several thousand participants, by five different teams of facilitators in locations sometimes only accessible by boat or plane, dissuaded the use of paper questionnaires. The first reason was logistical: paper-based data collection would result in additional handling and sorting, heavy weight of paper, and high risk of loss and damage during transportation by boat in the rain. A second reason was the complexity and highly variable length of questionnaires with many questions that would likely be skipped if using paper questionnaires. Use of paper would also have required extra time and staff for data entry, which would be incompatible with our need for swift access to data to monitor the safety of the study.

The device used for EDC needed to scan barcodes with a camera, and this determined the choice of tablets or smartphones. Tablets with a larger screen were preferred to allow facilitators to display videos, drawings, and a demonstration of the Malakit app during the training of participants [7,11]. The lack of stable and reliable internet access motivated the choice of offline data collection. At distant sites, electricity was only available in the evening for 4 to 5 hours with the use of a petrol generator. Upon choosing the electronic device for data collection, battery life was, therefore, a major selection criterion: the device had to have a battery life greater than 48 hours in case of prolonged energy shortage. Hence, a tablet with a 7-inch screen was selected for its maximum battery life of 100 hours: Galaxy Tab A 7.0 (model SM-T280, 2016, Android 5.1; Samsung) [12]. The advantage of a 7-inch versus a 10-inch tablet was also a lower weight, making it more ergonomic for a prolonged use at arm’s length. The selection of an Android device constrained the choice of the EDC app.

#### Data Collection and Storage

ODK (Open Data Kit) is an open-source ecosystem dedicated to data collection and management in challenging environments; it is actively developed and maintained by a worldwide community [13,14]. ODK Collect is an app that allows for offline data collection from mobile Android devices. Data may be encrypted and retrieved manually from the device or uploaded to a distant server implementing ODK Aggregate when internet access is available. ODK Collect met many requirements of the Malakit project, in particular, data encryption to comply with good clinical practice and the European Union’s General Data Protection Regulation, which are rigorous processes in the management of personal health data [15].

Deploying and maintaining an instance of ODK Aggregate on the study sponsor’s server was too expensive and complex for a single research project. Online services such as Ona and KoBoToolbox are alternatives to ODK Aggregate that were developed to assist humanitarian and research projects in low- and middle-income countries [16,17]. We selected Ona for its ability to manage several projects and allocate different user rights for form edition, data submission, and data access. Opting for a paid plan granted the capacity to handle sufficient numbers of weekly form submissions during the 2 years of the study; this also established a commercial bond securing data safety and server maintenance.

### Development of the Malakit Information System

**Figure 3:**
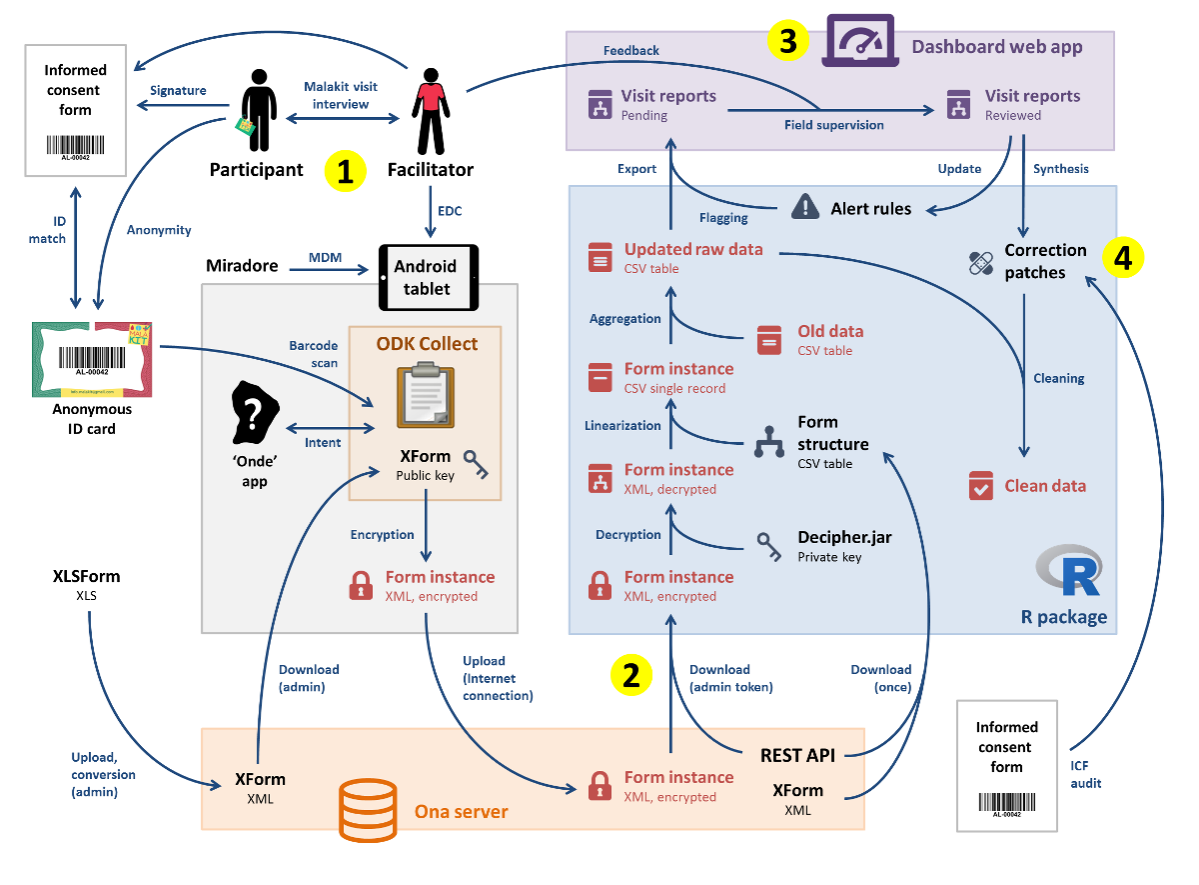
Information system of the Malakit study (collection and flow of data), Guiana Shield, April 2018-March 2020. (1) Mobile data collection by facilitators with Android tablets and storage on the Ona server. (2) Data retrieval, decryption, and aggregation with the MalakitR package. (3) Monitoring of visit reports with the Malakit dashboard web app. (4) Data cleaning with the MalakitR package. admin: administrator; EDC: electronic data capture; ICF: informed consent form; MDM: mobile device management; ODK: Open Data Kit; REST API: representational state transfer application programming interface; XForm: form standard used by ODK; XLS: Microsoft Excel spreadsheet.

The workflow of the final information system developed for the Malakit study is described in Figure 3.

#### Form Design for Electronic Data Capture

Malakit was designed as a longitudinal study with two different types of visits: a first inclusion visit to train the participants on the proper use of the kit, and subsequent follow-up visits to debrief participants on their episodes of symptoms of malaria and potential uses of the kit since their inclusion in the study [6,7]. Two main questionnaires were designed for the inclusion and the follow-up visits, respectively. The inclusion questionnaire covered sociodemographic data, mobility, gold mining activity, and the result of the malaria rapid diagnostic test (RDT) performed by the participants during their training. The follow-up questionnaire focused on malaria episodes experienced by participants since their last visit with a facilitator and their use of the kit. The main information collected was the severity of symptoms, the use of an RDT, the medication taken, the duration of treatment, and the experience of side effects. The follow-up questionnaire was more complex than the inclusion questionnaire because of the possibility of a participant to report up to five past episodes of symptoms of malaria, therefore requiring the repetition of related variables.

Drawing flowcharts for each questionnaire allowed for a first round of proofreading by explicitly representing skip logic and branching between questions (Multimedia Appendix 1). These also served as valuable references for testing and debugging the forms’ behavior in ODK Collect. Online form builders provided by ODK or Ona were not sophisticated enough to address the complexity of the Malakit questionnaires. In this case, documentation from ODK advises designing the form manually in a spreadsheet using the XLSForm (XLS: Microsoft Excel spreadsheet) standard [18,19]. We went a step further and developed a homemade XLSForm template that decouples the form’s content from its structure and logic. This facilitated modifications and the eventual translation of all text fields (ie, hints, warnings, questions, and choices for answers) into Portuguese, the main language spoken by the participants (Multimedia Appendix 2).

Malakit facilitators captured participants’ information using ODK Collect with the appropriate inclusion or follow-up questionnaire. On inclusion, the participants received a Malakit card with a barcode matching the anonymous ID number on their informed consent form (ICF); the barcode was then scanned with ODK Collect during the interview. In case participants returned for a follow-up visit without a card, facilitators filled in a confidential document with the name and date of birth of each participant and delivered a new anonymous ID number. Versions of ODK Collect ranging from version 1.14 (April 2018) to version 1.18 (November 2018) were used throughout the study. Critical features of ODK Collect, such as form record deletion, were restricted with an administrator password known only by the supervisors. Editing a record after closing and validating a form was impossible because of encryption.

#### Onde: A Custom App to Capture Names and Locations of Mining Sites

**Figure 4:**
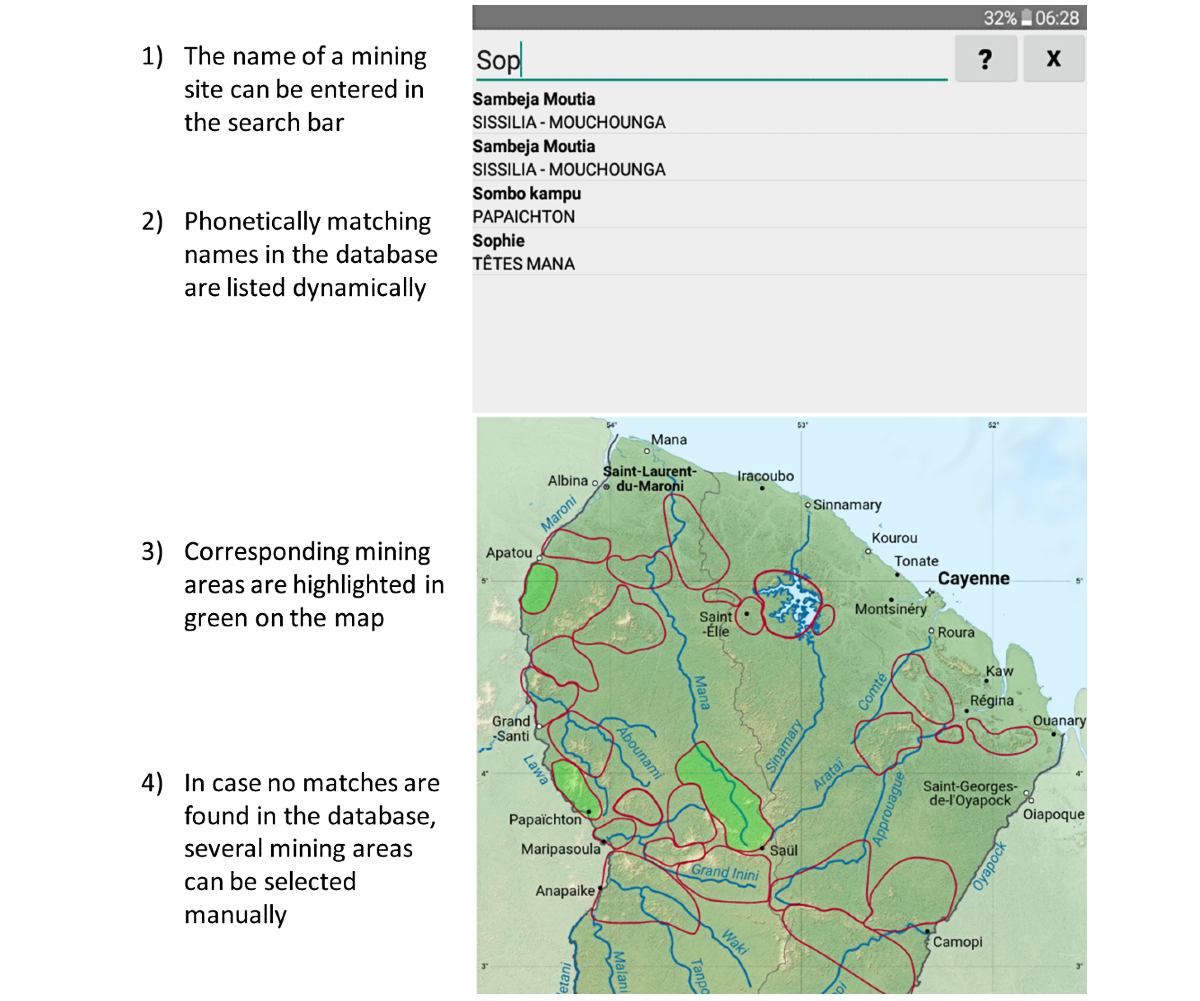
Screenshot of the Onde app (Malakit study, Guiana Shield, April 2018-March 2020).

Documenting the names and locations of mining sites was important for improving knowledge about the study population’s mobility and for identifying malaria hot spots. However, this was challenging since both participants and facilitators were not familiar with the geography of French Guiana and could use different names for the same site, with different possible spellings [5]. A simple, ad hoc Android app named Onde was developed with a database of names and coordinates of known gold mining sites curated by the Parc Amazonien de Guyane and a map of French Guiana displaying the main rivers, villages, cities, and main gold mining areas. This app aimed to improve user experience and data capture by implementing a phonetic-matching algorithm into an autocompleting search bar synced with an interactive map (Figure 4).

#### Data Retrieval, Decryption, and Aggregation: MalakitR Package

In order to streamline data retrieval and aggregation and allow continuous data monitoring, a custom MalakitR package was developed in-house to implement all steps in a single environment using R (versions 3.4.4 to 4.0.0; The R Foundation) and RStudio (versions 1.1.442 to 1.3.959) [20-22].

Encrypted data needed to be downloaded from Ona and then decrypted with ODK Briefcase [23,24]. However, the initial complexity of the follow-up visit questionnaire was improperly handled. In addition, the only solution to re-encrypt data that had been reshaped into an updated form structure and upload them to Ona was to manually re-enter the data into ODK Collect, since ODK Briefcase only handled decryption and not encryption. To implement form encryption and decryption, open-source Java code was extracted from ODK Briefcase (ie, decryption classes) and ODK Collect (ie, encryption classes) and compiled into a single executable decipher.jar file included in the MalakitR package [25,26].

The MalakitR package made it possible to download individual encrypted record files with the help of Ona’s application programming interface. Encrypted files that were newly downloaded were decrypted and linearized as single-record files according to the form structure; they were then finally aggregated with existing data to generate an updated table of raw data. During the study, all encrypted and decrypted data were securely stored on the study sponsor’s server in French Guiana. The source code of the MalakitR package is available on GitHub [20,25].

#### Data Monitoring, Validation, and Cleaning

Malakit data collected on the field needed to be monitored continuously in order to ensure their quality and to guarantee that the study protocol was respected. Any issues regarding the safety of participants were to be reported to principal investigators and to the study’s Data and Safety Monitoring Board. Because any difficulty or uncertainty was best debriefed with facilitators and local supervisors directly on the field, a simple portable and offline solution was needed.

A portable dashboard was developed as a JavaScript single-page app using the Vue.js framework (version 2.5) [27]. The MalakitR package included an additional set of functions to screen data records against alert rules defined by the coordination team, to detect data worth monitoring manually, and to generate visit reports that could be read inside the Malakit dashboard.

**Figure 5:**
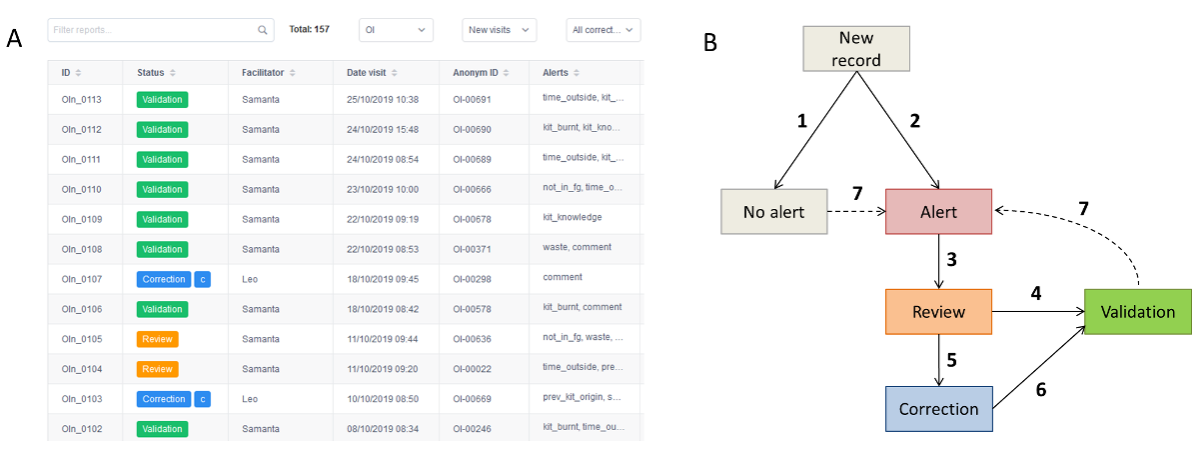
Data monitoring with the Malakit dashboard web app (Malakit study, Guiana Shield, April 2018-March 2020) A. Example of data records monitored. B. Flow of data reviewing and validation. Screened visit records are flagged with "No alert" (1) or "Alert" status (2), leading to manual review (3). After review and the facilitator’s feedback, records are validated (4) or flagged for correction (5) and patched (6). Records can be screened against new alert rules (7).

In the dashboard web app, visit reports displayed pairs of variable names and values in a user-friendly structure. Variables flagged with alerts were highlighted in red to facilitate the review of the data and consequent decision-making. Figure 5 shows a screenshot of the dashboard main screen and the progression of new visit records through the different statuses of the data monitoring process.

#### Training and Supervision of Facilitators

Practical training sessions were organized throughout the deployment phase of the project. Nine facilitators were trained to fill in the ICF, to assign a new anonymous ID number to a participant, to use Android tablets, to enter data during mock visit interviews with ODK Collect and the Onde app, to upload data using a Wi-Fi network, and to ensure the successful transfer of all pending form records. Training tablets configured with a “sandbox” Ona account were used for this purpose.

Facilitators were supervised on the field at the opening of a new distribution site. Tablets were provided with foldable protective covers and waterproof bags for storage and transportation. Inclusion and follow-up visit forms were specific to each distribution site. This allowed for getting quick activity feedback on the number of forms uploaded from each distribution site directly from Ona. This also avoided multiple updates of the forms in case of facilitator turnover at a single distribution site. Subsequent supervision visits were carried out regularly at all distribution sites by members of the Malakit coordination team and field supervisors. Feedback from facilitators was collected in a nonstructured way throughout the Malakit study during field visits and WhatsApp conversations. At the end of the study, seven facilitators answered a series of questions about their whole experience in the study in the form of short videos, which were displayed during the final meeting of the Malakit project in October 2020 [28].

### Ethics Approval

The Malakit study obtained all competent ethical approval required according to the respective regulations in Brazil and Suriname: the ethical committee of the Fundação Oswaldo Cruz in Brazil (CAAE [Certificado de Apresentação de Apreciação Ética] No. 89482118.0.0000.5248; approval No. 2.831.534) and the ethical committee in Suriname (CMWO [Commissie voor Mensgebonden Wetenschappelijk Onderzoek] approval No. VG 25-17). Written consent was obtained from all participants. This study was registered at ClinicalTrials.gov (NCT03695770).

## Results

### Overview

From April 2018 to March 2020, nine facilitators generated a total of 4863 form records at the five distribution sites of the Malakit project. Herein, we present feedback results from study facilitators and data monitoring by the coordination team.

### Feedback From Facilitators

Facilitators’ feedback during training and supervision visits at distribution sites was valuable for adapting the information system once the study was fully implemented.

#### Adaptations Made After Facilitators’ Training and Study Launch

Using the tablet, switching Wi-Fi on and off, playing training videos, and using ODK Collect was straightforward enough, since most facilitators were already familiar with Android devices. Removing the answer from an already-completed field in ODK Collect was not intuitive because it required a long press on the field to erase. Learning to use the Onde app was a difficult step of the training for some facilitators who were unfamiliar with the geography of French Guiana or the names of mining sites; however, the facilitators found the interface of the app intuitive enough.

Some adaptations were made to improve the facilitators’ work: installation of a specific keyboard app for one facilitator with sight impairment, use of capital letters instead of digits for months when writing down dates of visit and dates of birth on paper ICFs, and additional practical training for older facilitators who were less experienced in using tablets.

Facilitators were able to clearly explain the context and objectives of the Malakit study to the gold miners, and obtaining their written consent was not an issue. At first, filling in the ICFs correctly with participants was more challenging for facilitators without administrative experience in research or health work. With a little practice, this task quickly became routine, and ICF audits were satisfactory. Facilitators initially confused follow-up visits with inclusion visits on several occasions; this required important clarifications about the difference between the data collected for each type of visit and their purpose in the evaluation of Malakit.

#### Technical Difficulties

The coordination team in Cayenne provided facilitators with assistance through instant messaging on WhatsApp. Facilitators sent pictures or videos to demonstrate the problem, thereby improving its analysis and resolution. Several technical problems were reported by the facilitators (Table 2), but no big failure of EDC was experienced on the field during the 2 years of the study. In particular, facilitators took great care of the tablets and none were lost, stolen, or damaged, nor showed eventual battery issues.

**Table 2:** Technical issues with electronic data capture reported by facilitators during the Malakit study, Guiana Shield, April 2018-March 2020.

Category and problem		Frequency	Note	Solution
**Hardware**				
	Loss or damage of the cable and charger of the tablets	Frequent	This happened at all distribution sites.	These were easy to replace with a compatible USB charger and cable.
	Slight loss of contrast on the edges of the screen of the tablets	Twice (end of the study)	This was observed at the distribution sites with the highest humidity.	This did not alter the proper functioning of the tablets.
	Fear of losing the tablet, leading to use of a blank piece of paper and postponed data entry	Once	This was detected in the dashboard because the kit ID was consistently entered manually and visits were concentrated within a short period of time at the end of a workday.	The facilitator was reassured and asked to use the tablet to avoid errors.
**Data entry**				
	Forgetting to close and validate the form in ODK^a^ Collect right at the end of the visit	Frequent (start of the study)	This resulted in a wrong end time stamp and an abnormally long duration of the questionnaire.	Facilitators were shown how to ensure that a form was closed and encrypted. When necessary, data were retrieved manually.
	Difficulty scanning kit barcodes in dim light, leading to manual data entry	Frequent	The transparent labels with the kit barcode were pasted on the pink cover of the medication pouch to facilitate the identification of kits, but this resulted in a lower contrast when scanning.	Facilitators were offered to paste the barcode label on a white area inside the kit, at the expense of having to open the kit to scan and identify its ID during inventory.
	Scanning barcode of a kit ID instead of an anonymous ID, which blocked the form progression	Once	The form progression was blocked because an incorrect ID format was detected by the regular expression validation constraint. The facilitator simply started again with a new form.	Facilitators were reminded of how to remove an answer in ODK Collect with a long press.
**Tablet configuration**				
	After a prolonged period without use, one tablet was completely discharged and the date was set to the year 1923, blocking the form progression	Once	The form progression was blocked because the automated calculation of the participant’s age based on date of birth and the year 1923 returned a negative value.	Facilitators were asked to check that the date of the tablet was correct before launching ODK Collect, especially after a long period without use.
	Incorrect time in the tablet due to wrong time zone setting	Once	The time in Suriname, French Guiana, and the North Region of Brazil is GMT–3 throughout the year; in the tablets, this coincided with Buenos Aires instead of Brasilia time, which had daylight time change in February and November (until 2019).	Facilitators were asked to change the time zone setting of the tablet.

^a^ODK: Open Data Kit.

### Feedback From Data Monitoring

Data were downloaded and monitored on a weekly basis. WhatsApp messaging was, again, the fastest way of getting feedback from facilitators, but in-depth debriefing was only possible in a face-to-face context on the field. Sometimes a lag time elapsed between data monitoring and actual field supervision, resulting in memory issues when discussing with facilitators. Once facilitators validated a form record, encryption prevented them from looking at the record and editing the data. In case of doubt regarding the data entered for a particular visit, facilitators were asked to warn the coordination team about the suspected issue for verification. The dashboard was a good tool for visualizing all the information of a visit at once and for guiding the debriefing with facilitators. Supervision visits were more frequent during the months following the opening of a new distribution site or when training of a new facilitator was necessary.

From April 2018 to March 2020, facilitators from the Malakit project generated a total of 4863 form records, corresponding to 3897 inclusion visits (n=2454, 62.97% from Suriname and n=1443, 37.03% from Brazil) and 966 follow-up visits (n=709, 73.4% from Suriname and n=257, 26.6% from Brazil). This corresponded to an average of 202 record submissions per month to the server. Failure to send data was very rare (42/4863, 0.86%): 35 records (0.72%) were retrieved manually from the facilitators’ tablets during regular supervision visits at the distribution sites and 7 (0.14%) could not be retrieved. Duplicate records were found for 7 (0.14%) visits. A final number of 4849 unique visits were exploitable with complete data (n=3888, 80.18% inclusion visits and n=961, 19.82% follow-up visits).

**Figure 6:**
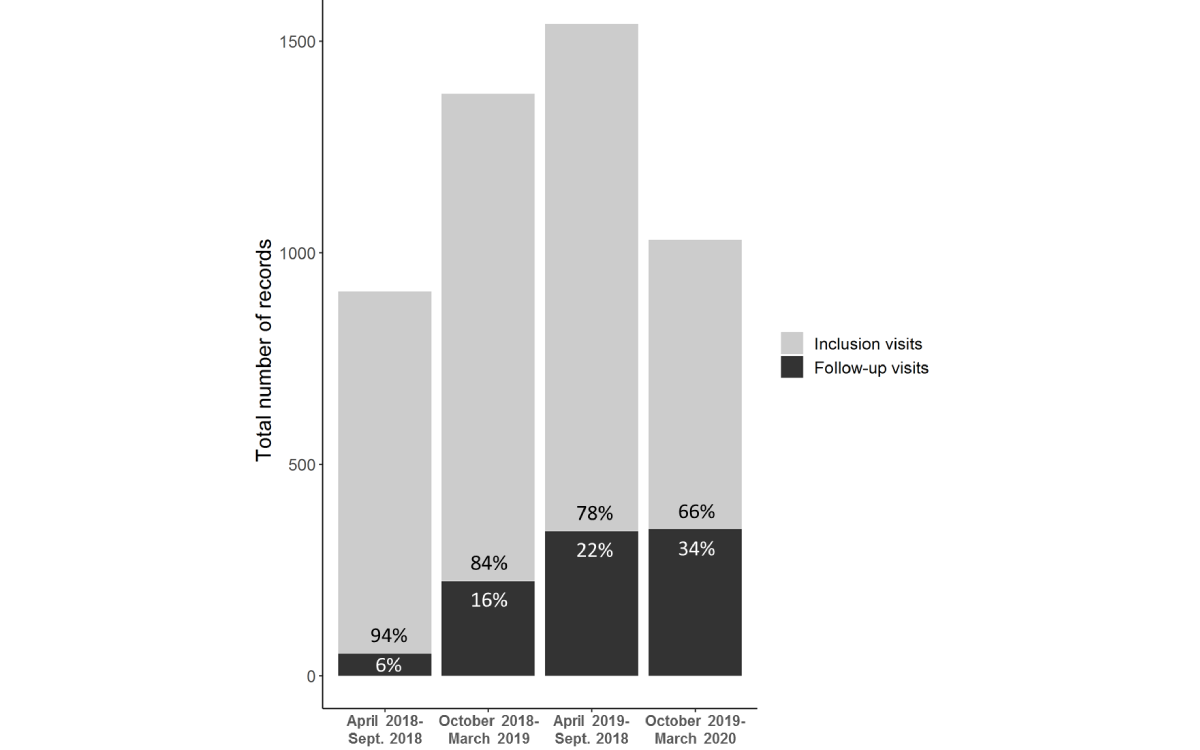
Number and proportion of records for inclusion and follow-up visits according to the 6-month period of study (Malakit study, Guiana Shield, April 2018-March 2020).

Follow-up visits were rare during the first 6 months of the study, but their frequency increased steadily over the following 6-month periods (Figure 6). This was expected, given the median length of stay on mining sites of about 3 months [3]. Sufficient feedback on the follow-up visits was obtained between July and November 2018 to develop a better follow-up questionnaire and a methodology to structure the visit process. The new version of the questionnaire was implemented at all distribution sites in January 2019 during two group training sessions of facilitators. Following this major change in the structure of data, 161 existing follow-up visit records were converted, encrypted, and reuploaded to Ona to ensure the centralization of data and the consistency of the total number of form records submitted.

**Figure 7:**
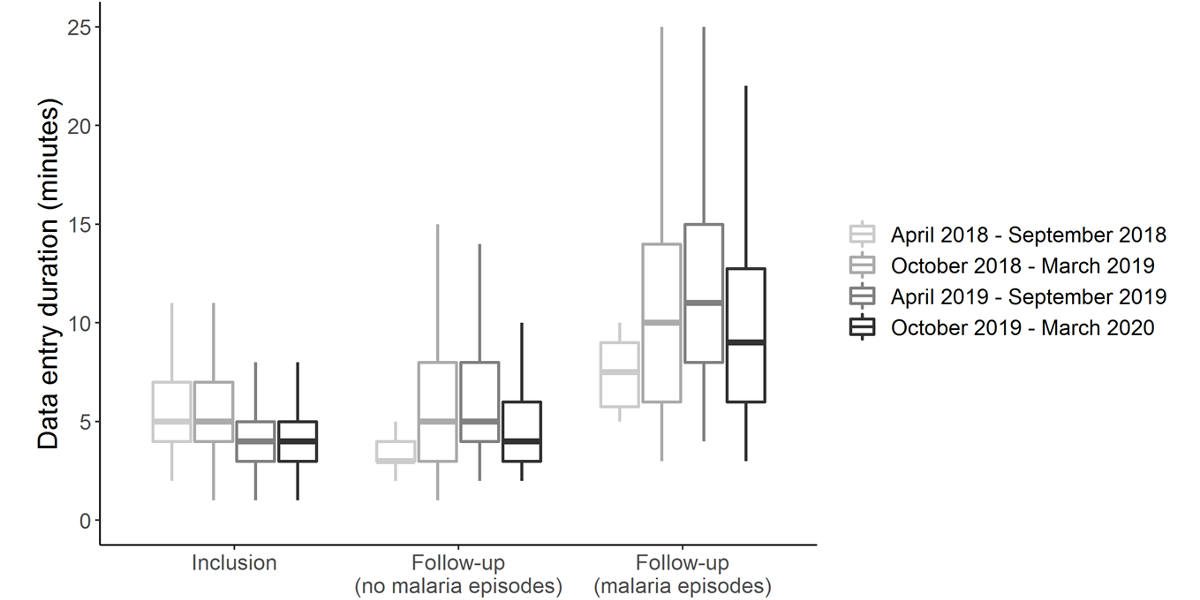
Duration of data entry for the inclusion and follow-up questionnaires, with and without malaria episodes reported by participants (Malakit study, Guiana Shield, April 2018-March 2020). The horizontal lines within the boxes represent medians and the whiskers represent IQRs.

Data capture with ODK Collect was swift according to the record metadata: the median duration was 5 (IQR 3-7) minutes (n=4856), with both types of visits combined. Given that visits usually lasted an estimated total of 30 to 45 minutes, this suggests that EDC was not taking more time from the training or debriefing of participants. A minority of 1.32% (64/4856) of the durations exceeded 60 minutes, usually because the form records were not closed and validated immediately at the end of an interview. For inclusion questionnaires, a median duration of 4 (IQR 3-6) minutes (n=3892) was measured, with a median number of 27 (IQR 26-28) variables completed. The median duration was similar for the follow-up questionnaire when the participants did not report malaria episodes: 5 (IQR 3-7) minutes (n=611) for a median number of 24 (IQR 23-25) variables completed. The follow-up questionnaire became lengthier when participants reported at least one episode of malaria: median duration was 10 (IQR 7-15) minutes (n=350) for a median number of 46 (IQR 41-50) variables completed. Figure 7 shows that the duration of the questionnaires decreased over the course of the study. This was especially true for inclusion visits: 62.02% (1246/2009) versus 34.09% (642/1883) of inclusion questionnaires lasted more than 4 minutes during the first and second versus third and fourth 6-month periods of the study, respectively (*χ*^2^_1_=303.5, *P*<.001). This suggests that facilitators experienced a learning curve while using ODK Collect.

Over the course of the study, 18.31% (712/3888) of inclusion visits and 90.6% (871/961) of follow-up visits raised alerts; the discrepancy results from the greater number of alerts configured specifically for follow-up visits due to their higher complexity. A large proportion of follow-up visits (381/961, 39.6%) included written comments from facilitators. This confirmed the relevance of this feature to collect facilitators’ feedback and improve the quality of data collection and supervision. In total, 14.23% (690/4849) of visits raised alerts related to data entry or data accuracy. The most common alerts for data entry were manual barcode entry (158/4849, 3.26%), visit duration longer than 60 minutes (112/4849, 2.31%), and incoherent date of visit (34/4849, 0.70%). An important proportion of visits required feedback from facilitators either from WhatsApp or field supervision visits: 20.4% (145/712) of inclusion visits and 32.0% (279/871) of follow-up visits with alerts. Corrections were made to the data for 3.03% (147/4849) of the total visits, more frequently for follow-up visits (108/961, 11.2%) than inclusion visits (39/3888, 1.00%). Problems with data quality or protocol compliance were more frequent at the two most remote distribution sites. This could be linked to the higher difficulty for the coordination team to travel to these sites on a regular basis.

In 37.7% (362/961) of follow-up visits, participants were able to show an anonymous ID card. At the end of the ICF audits, 52 out of 3888 (1.34%) inclusion visits were found without a documented ICF, mainly owing to the loss of the last batch of 50 paper-based ICFs from Brazil at the end of the study. Although unfortunate, the removal of associated data was of limited impact because it mostly concerned participants included at the end of the study and very unlikely to attend a follow-up visit.

Comparison of dates of birth of the participants both written in the ICFs and entered in ODK Collect by facilitators provided insight into the consistency of data entry. Among the 3777 ICFs with a known date of birth (52 missing and 59 with an estimated age only), 113 (2.99%) mismatches with form records were identified, 73.5% (83/113) of which were minor differences, such as an increment or a decrement of 1 year.

## Discussion

### Adapt and Reuse; Do Not Reinvent the Wheel

The information system of the Malakit study is a patchwork of existing open-source services (ie, ODK Collect and R packages) and commercial services (ie, Ona) as well as tools developed in-house, of which some are innovations and may be adapted to other contexts beyond interventional health research. In particular, we developed an Android app compatible with ODK Collect as a means to provide a dynamic user interface and improve the collection of fuzzy geographical data. We also developed an R package allowing data managers to retrieve and clean data captured with ODK Collect and that supports bidirectional encryption and decryption.

The development of the Malakit information system was guided by the needs and constraints of the project. According to the MDC manual of the US Global Development Lab, the design and setting of the Malakit study fit into the highly complex category for all three dimensions examined: survey, analysis, and local context [29]. In this situation, the manual advises the hiring of an external consultant or the development of internal capacity. In order to better adapt to the complex and iterative implementation of the project, we decided that the development of the information system and the data management would rely on in-house human resources and technical skills. We chose, however, to contract with an external company to ensure the quality and compatibility of the Malakit smartphone app, which was designed as an additional evaluation tool of the Malakit project [6]. This app can be considered as a separate information system and mHealth intervention; its complete description and evaluation will be the object of a separate paper.

Our strategy was to reuse existing mature technology and fill the gaps with custom and retro-engineered components adapted to specific needs. In the event that in-house development of new tools is an option, as in this project, one should be careful not to reinvent the wheel and should consider existing tools and focus on developing tools specifically tailored to the needs of the project [30,31]. In that respect, mature open-source ecosystems, such as ODK, allow for a trade-off between innovation, adaptation, and reuse of existing solutions. ODK Collect, along with ODK Aggregate and ODK Briefcase, has been widely used in other settings and modified to answer particular needs in some studies [32-38]. The use of R or Stata downstream of ODK has been implemented by other research teams with success; the MalakitR package is another contribution to this open ecosystem [32,39]. The portable dashboard was a prototype that will not be maintained because it relies, in part, on deprecated technology. However, it helped specify requirements for the external development of a similar tool for future field studies.

Safety and confidentiality of personal data are essential and should dissuade nonprofessional developers to build their own system from scratch, especially when online transfer of health-related data is at stake [40,41]. Open or interoperable alternatives to ODK, such as REDCap (Research Electronic Data Capture), Enketo, CommCare, or OpenMRS (medical record system), may be worth considering for data collection and monitoring [31,42-45]. No unique EDC solution would fit all the needs of a study; some authors even recommend using several EDC tools when relevant [46]. Selecting components of an EDC system is not easy, and both field experience and structured guidance are valuable [29,47,48]. Provided that frequent updates are made to keep up with the rapid evolution of technology, online decision tools, such as NOMAD (Humanitarian Operations Mobile Acquisition of Data) or Kopernik, may help in specifying one’s needs and in finding an appropriate solution [49,50].

### Strengths and Weaknesses: Lessons Learned From the Field

Our experience is another contribution to the evidence reported in the literature, in that health mediators can successfully collect complex data using MDC tools when provided with adequate training and supervision [51]. Training facilitators, first in groups and then individually on the field, proved beneficial to better adapt the MDC strategy and increase their confidence with the technology they used. This experience is in line with the conclusions and recommendations from other studies [31,36,38,52,53]. Field staff turnover occurred at several distribution sites during the 2 years of the study and required timely training sessions, including, but not limited to, data collection. On one occasion, the training was successfully delivered by an already-experienced facilitator, allowing the coordination team to later focus on building capacity on specific points in the questionnaires.

The information system was reliable and the quality of the data collected by facilitators was satisfactory, allowing for a robust analysis of the main results of the study [54]. The choice of tablets was eventually strengthened by the absence of loss, damage, or battery failure, unlike that experienced in other studies [39]. A 7-inch screen, larger than a smartphone, allowed for the display of training material and was of great help for one sight-impaired facilitator, as reported by Dickinson et al [55]. Few technical issues were reported, few visits failed to be sent to the server, and virtually no visits lacked exploitable data. A significant number of visits required feedback from facilitators, but very few resulted in data correction. The audits of the ICFs found limited and minor mistakes in the data entry of dates of birth. Remote and field debriefings with facilitators aided by a portable dashboard web app greatly improved the monitoring of data and the detection of protocol issues or difficulties with the questionnaires. Feedback from the first follow-up visits helped bring major modifications to the follow-up questionnaire with a quick implementation, resulting in the integration of free comments from facilitators, which, in turn, helped with debriefing and contextualizing their issues.

Form testing is an essential precaution noted by other authors [39,46,52,56]. Knowing that losing any of the gold miners’ time would be detrimental to their participation in the study, we put a lot of effort into testing and adapting the electronic forms according to the facilitators’ feedback. This optimized the forms’ design and usability. Representing the branching logic of questions with a flowchart was useful for clarifying the expected electronic behavior of questionnaires that were drafted on paper and for guiding test simulations. Building an XLSForm template also sped up translations and modifications of the form. All of these efforts contributed to the short duration of data entry by facilitators, as shown by the analysis of the form metadata generated by ODK Collect. This freed up time for the actual training or debriefing of participants during visits.

The advantages of EDC over paper questionnaires are well established, especially in the case of complex studies [34,55,57]. EDC suppresses the need for long and error-prone data entry and, thus, shortens the delay between data collection and analysis [58]. However, EDC may in some situations raise issues of trust between data collectors and participants [35,58]. In Malakit, the careful hiring of facilitators from the population of interest helped in that regard; trust of participants in both the facilitators and the investigators was key to the success of the intervention [7]. Still, paper was used to collect informed consent and was, thus, not totally eliminated from the Malakit study. The loss of one batch of 50 ICFs illustrates the hazards associated with a fully paper-based system, especially in a complex setting such as ours. Although the removal of associated data had no impact on the outcome measure (ie, correct kit use) and virtually all ICFs were retrieved and audited successfully, electronic alternatives may be worth implementing for future projects [59,60].

The use of anonymous ID cards was not efficient. Few participants came back with their card, and mix-ups between participants who had attended the same inclusion visit were occasionally detected in the data. Participants of the study trusted the facilitators with their identity and did not have a problem disclosing it again when they could not show a card. Such challenges in identifying study participants from hard-to-reach populations have been reported with ID cards [61]. Alternatives such as fingerprints or photographs may be considered, but their acceptability in the gold miner population would require careful participatory evaluation and validation, as well as ethical and regulatory clearance [32,61].

### Perspectives

The different tasks associated with the Malakit information system fit into a continuum between digital tools development, field supervision, and data management, which all require different levels of technical skills and time resources. These tasks could be achieved by a single person during the 2-year period of the study, in part owing to the low volume of data generated in our study when compared with other studies: less than 4000 participants were included in Malakit versus 25,000 and 280,000 participants according to Style et al and Marks et al, respectively [32,39]. For such larger studies, a team dedicated to data management, supervision, and system development should be set up [41,45]. One lesson learned from the field was the need for local supervisors fully dedicated to the research project. A solution to maintain more constant and reactive data supervision would be to delegate part of the process to local supervisors through capacity building [58].

Strategies to improve data collection directly from the mining areas could be evaluated using text messaging, instant messaging, or ODK Collect with Bluetooth data transfer [38,62]. The dashboard web app used for the Malakit study was a prototype that would only suit the specific needs of the supervision team. A new dashboard developed as a stand-alone Shiny app would better interface with the MalakitR package [63]. Setting up our own instance of ODK Central would help in complying more easily with French and European regulations regarding data protection and in using the R package ruODK as an interface [32,64]. In the long run, ODK-X offers interesting perspectives for the management of longitudinal data [65,66].

The components of the Malakit information system can be useful as a whole or in part for research teams wishing to collect and monitor encrypted data in similar conditions as in our study: (1) remote inclusion sites with little access to internet and electricity, (2) data collected by people without experience in research, and (3) continuous data monitoring and quality control. The system can be implemented and managed by personnel with sufficient computer skills and knowledge of R, in close coordination with field workers and supervisors.

The information system described in this paper evolved into a simplified version for monitoring Malakit as a public health intervention in Suriname. It may contribute to a coherent and coordinated public health action in the region and eventually interface with existing cross-border surveillance systems of malaria [67].

### Conclusions

The development of the information system for the Malakit project was a source of innovation that mirrored the inventiveness of the intervention itself. Unprecedented in this particular context, the development had to adapt iteratively to the constraints and different phases of the study. Some of the components are available for reuse in different research contexts. Our experience with the Malakit information system confirms that, provided that adequate care is brought to design, training, and supervision, health mediators are able to capture good-quality data to support the evaluation of a complex research study in a challenging environment.

## Appendix

Multimedia Appendix 1 Flowchart showing skip logic and branching of inclusion questionnaire.

Multimedia Appendix 2 Template XLS form used for inclusion questionnaire. XLS: Microsoft Excel spreadsheet.

## Abbreviations

CAAE: Certificado de Apresentação de Apreciação Ética
CC BY-SA 3.0: Creative Commons Attribution-ShareAlike 3.0 Unported
CC BY-SA 4.0: Creative Commons Attribution-ShareAlike 4.0 International
CMWO: Commissie voor Mensgebonden Wetenschappelijk Onderzoek
EDC: electronic data capture
ICF: informed consent form
MDC: mobile data collection
mHealth: mobile health
MRS: medical record system
ODK: Open Data Kit
RDT: rapid diagnostic test
REDCap: Research Electronic Data Capture
XLS: Microsoft Excel spreadsheet
XLSForm: form standard used for the authoring of forms in a spreadsheet
