# Supplementary figures and images for: Tailoring Mobile Data Collection for Intervention Research in a Challenging Context: Development and Implementation in the Malakit Study
Source: JMIR Form Res. 2022 Jun 16;6(6):e29856. doi: 10.2196/29856 (PMC9247814; doi:10.2196/29856)

12/06/2018

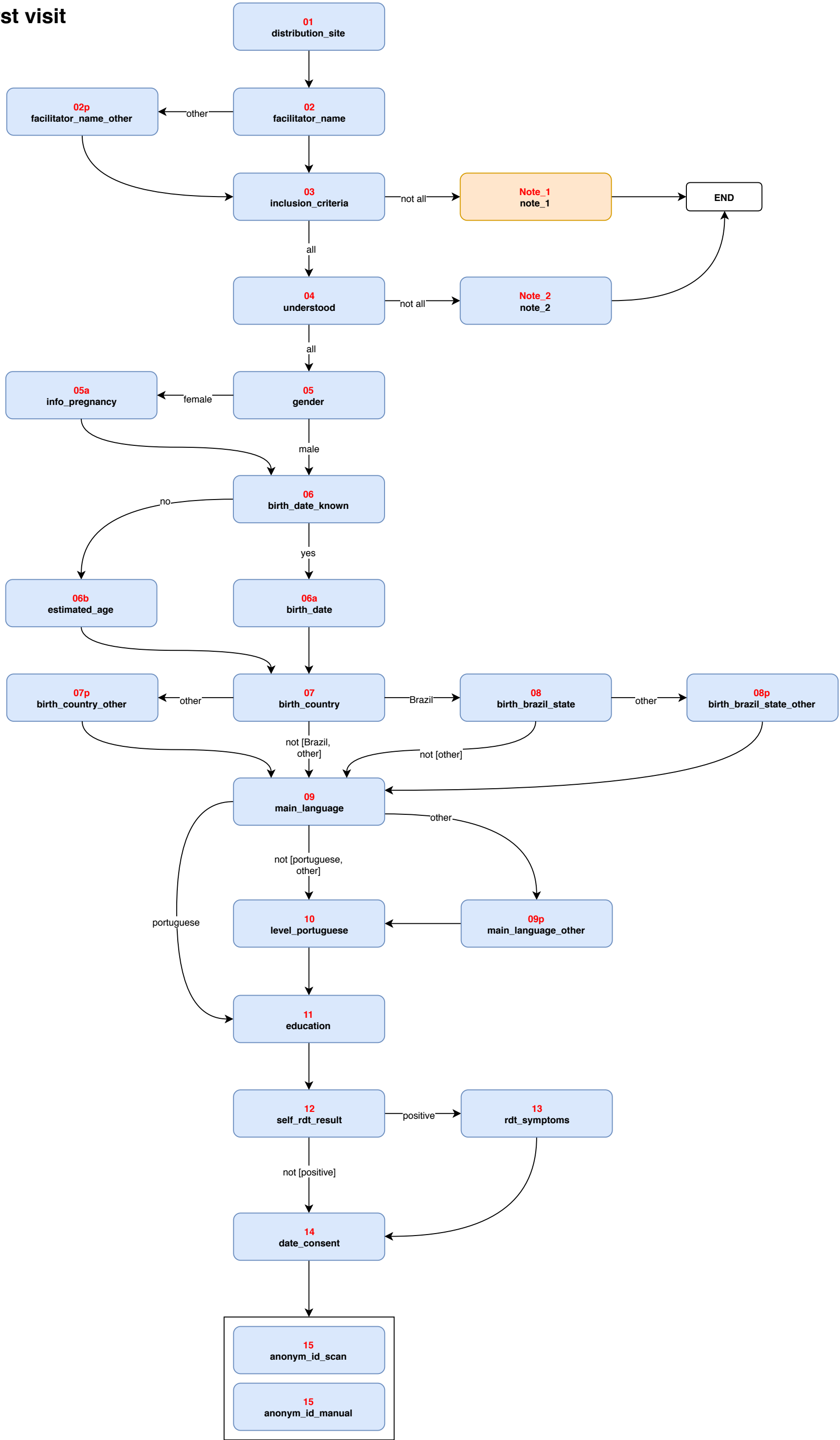

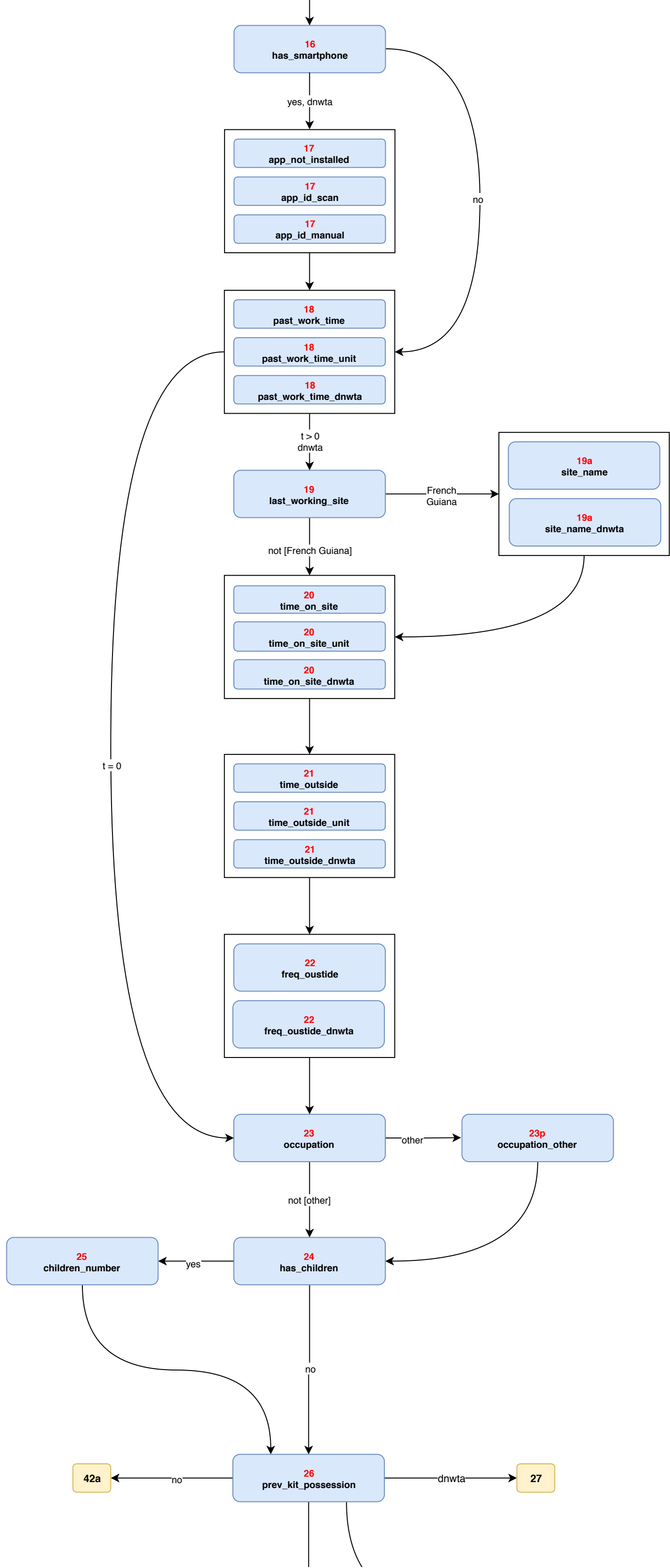

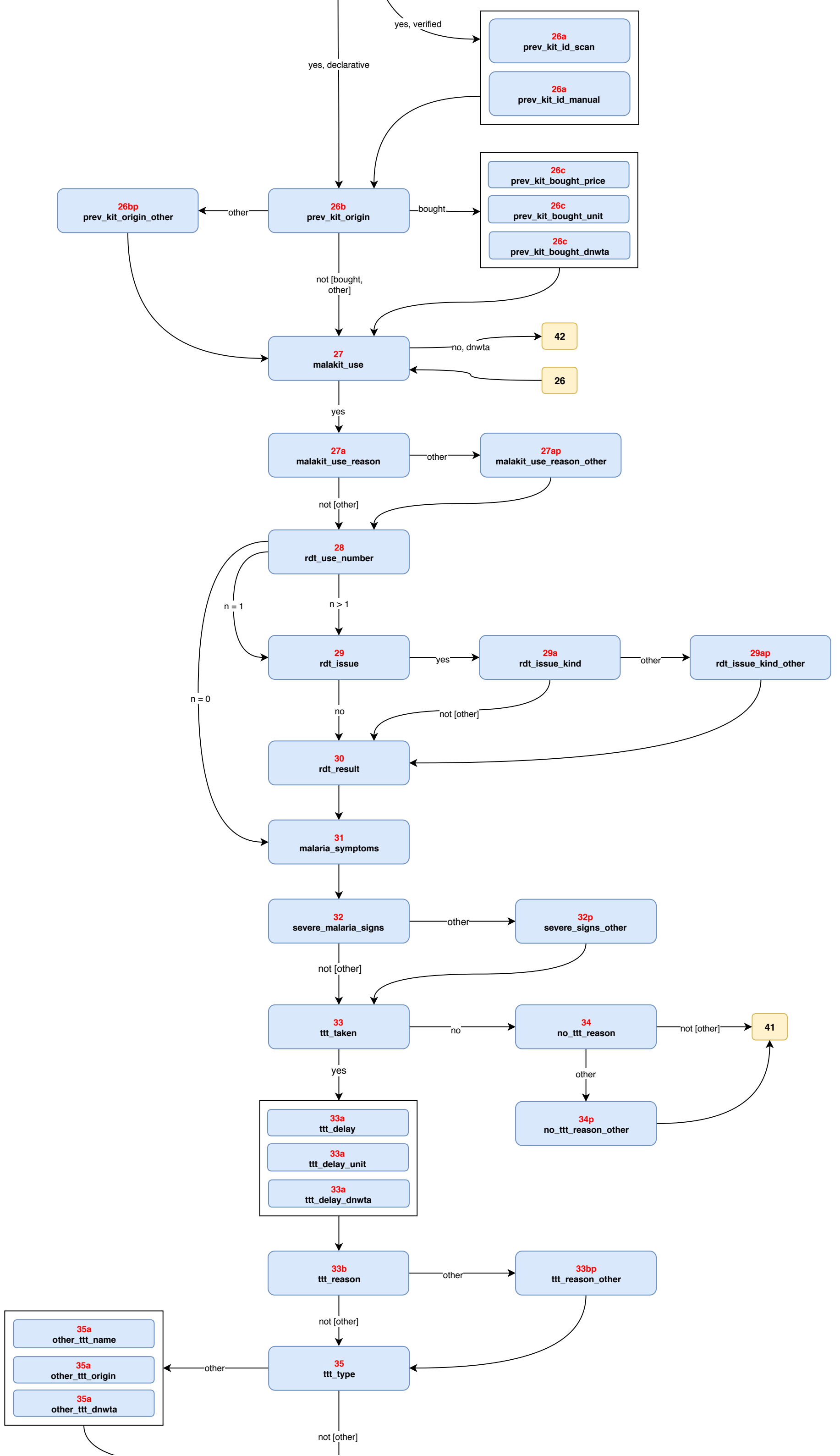

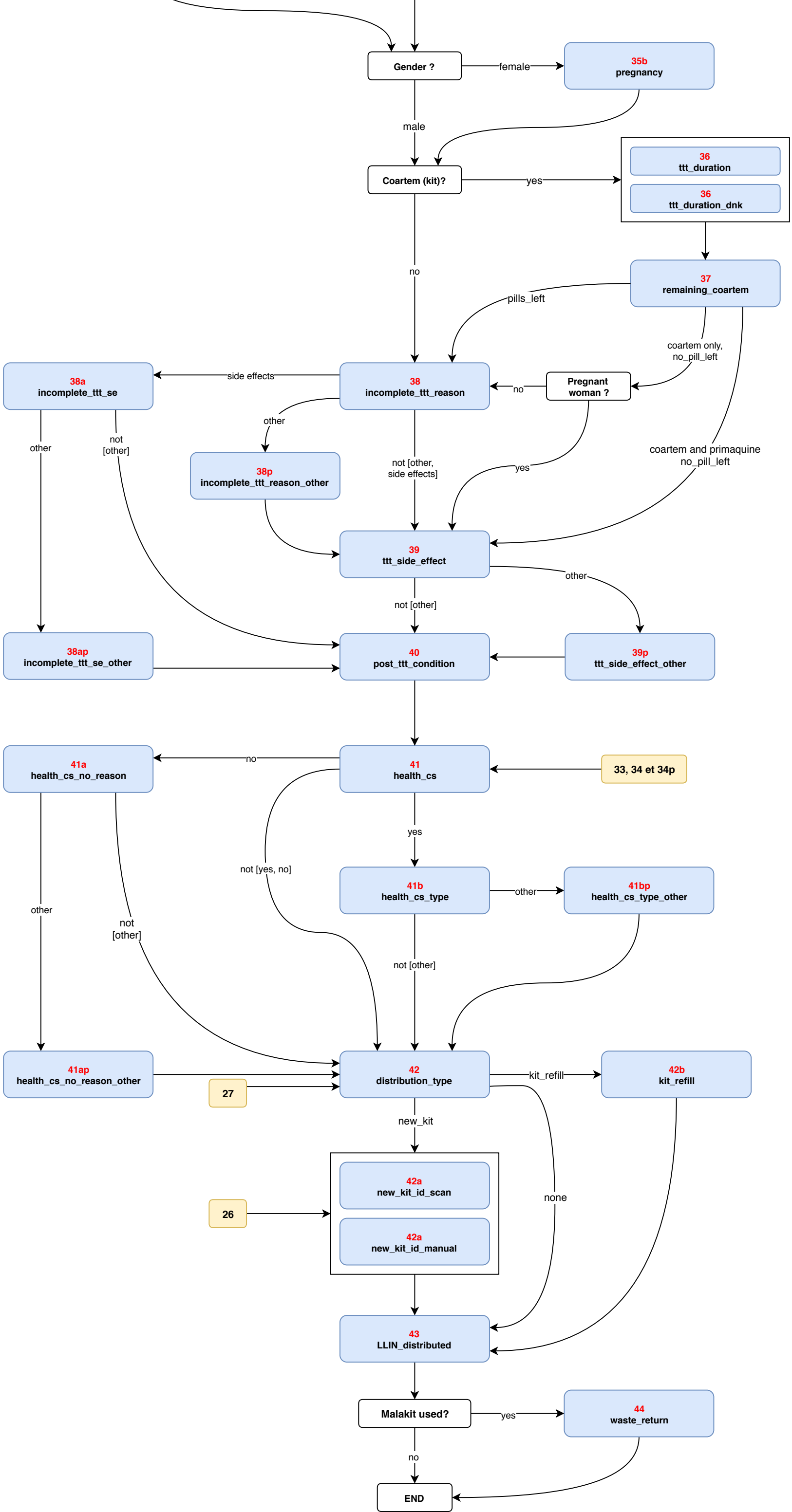

Supplement: Multimedia Appendix 1 [file formative_v6i6e29856_app1.pdf]
